# Randomized trial of nirmatrelvir/ritonavir versus placebo for adults with acute COVID-19 to prevent long COVID: PanoramicNOR Trial

**DOI:** 10.1186/s13063-025-09226-6

**Published:** 2025-11-06

**Authors:** Bjørn Blomberg, Nikolai Norevik Myklebust, Oddvar Oppegaard, Camilla Tøndel, Rebecca Jane Cox, Arild Iversen, Marianne Emblem Lehmann, Axel Sandvig, Tuva Børresdatter Dahl, Vibeke Devold Valderhaug, Peter Szodoray, Maja Wilhelmsen, Bjørn Eivind Kirsebom, Marte Glambek, Oddvar Kaarbøe, Pål Aukrust, Rolv Terje Lie, Nina Langeland

**Affiliations:** 1 Department of Clinical Science, University of Bergen, Bergen, Norway; 2 National Centre for Tropical Infectious Diseases, Department of Medicine, Haukeland University Hospital, Bergen, Norway; 3 Department of Medicine, Haukeland University Hospital, Bergen, Norway; 4 Department of Paediatrics, University of Bergen, Bergen, Norway; 5 Influenza Center, Haukeland University Hospital, Bergen, Norway; 6 Chief Municipal Doctor’s Office, Bergen Municipality, Bergen, Norway; 7 St. Olav’s Hospital, Trondheim, Norway; 8 Norwegian University of Science and Technology, Trondheim, Norway; 9 Research Institute for Internal Medicine, Oslo University Hospital, Rikshospitalet, Oslo Norway; 10 Department of Research and Innovation, Møre Og Romsdal Hospital Trust, Ålesund, Norway; 11 Department of Neuromedicine and Movement Science, Faculty of Medicine, Norwegian University of Science and Technology, Trondheim, Norway; 12 Department of Immunology, Oslo University Hospital, Rikshospitalet, Oslo Norway; 13 University Hospital of Northern Norway, Tromsø, Norway; 14 Arctic University of Norway, Tromsø, Norway; 15 Department of Neurology, University Hospital of North Norway, Tromsø, Norway; 16 Department of Psychology, Faculty of Health Sciences, The Arctic University of Norway, Tromsø, Norway; 17 Department of Global Public Health and Primary Care, University of Bergen, Bergen, Norway; 18 Institute of Clinical Medicine, University of Oslo, Oslo, Norway

**Keywords:** SARS-CoV-2, COVID-19, Post-COVID-19 condition, Long COVID, Prevention, Randomized controlled trial, Paxlovid, Nirmatrelvir/ritonavir

## Abstract

**Background:**

The high prevalence of long-term persisting symptoms after COVID-19 (coronavirus disease 2019), termed long COVID or post-COVID-19 condition, even among those with mild initial disease, may have a large public health impact. Apart from avoiding infection, there is no proven prevention or treatment for long COVID. We will perform a randomized placebo-controlled clinical trial to assess whether treatment with the novel antiviral, nirmatrelvir and ritonavir (Paxlovid®) for acute COVID-19 can prevent the development of long COVID.

**Methods:**

This is a randomized double-blinded placebo-controlled trial that aims to recruit 2000 nonpregnant persons aged 18 to 64 years with acute COVID-19 with positive PCR and/or antigen test and symptom duration of not more than 5 days. Participants will be randomized 1:1 to a 5-day course of Paxlovid (two tablets 150-mg nirmatrelvir and one tablet 100-mg ritonavir twice daily) or a 5-day course of placebo (similar number of tablets of equal appearance). The primary endpoint will be persistent symptoms compatible with long COVID, assessed as the prevalence of a dichotomous variable corresponding to the presence (1) or absence (0) of one or more of the following symptoms: (i) fatigue, (ii) dyspnea, and (iii) cognitive symptoms (memory and/or concentration problems). The primary outcome will be evaluated at 3-month follow-up and then re-evaluated at 6, 12, and 24 months.

**Discussion:**

As more than 750 million people with confirmed COVID-19 have survived globally, the potential burden of long COVID on societies is formidable. If a simple 5-day oral treatment course with nirmatrelvir/ritonavir is shown to prevent long COVID, it would be a highly attractive intervention at an individual level and a mitigation of its public health consequences.

**Trial registration:**

ClinicalTrials.gov NCT05852873. Registered on May 2023.

**Supplementary Information:**

The online version contains supplementary material available at 10.1186/s13063-025-09226-6.

## Administrative information

**Table:** 

Title {1}	Randomized trial of nirmatrelvir/ritonavir versus placebo for adults with acute COVID-19 to prevent long COVID: PanoramicNOR Trial (Platform Adaptive trial of NOvel antiviRals for eArly treatMent of COVID-19 In the Community in Norway)
Trial registration {2a and 2b}	Registered in clinicaltrials.gov on 10th May 2023 with ID NCT05852873. Registered with EudraCT with ID 2022–003244-27. Registered in CTIS with ID 2023–510337-29–00. Approved by the Regional Committees for Medical and Health Research Ethics of South-East Norway with ID REC-SE-578741
Protocol version {3}	Based on protocol version 2.3 (2023.03.23) and statistical analysis plan version 1.0 (2023.05.10)
Funding {4}	• KlinBeForsk grant no. 34476 • Western Norway Regional Health Authority, grant no. F-12621 • Helse Møre og Romsdal Hospital Trust, grant no. 2024/2058 • The Influenza Center, Haukeland University Hospital, University of Bergen
Author details {5a}	Bjørn Blomberg, Department of Clinical Science, University of Bergen, National Centre for Tropical Infectious Diseases, Department of Medicine, Haukeland University Hospital, Bergen, Norway. ORCID 0000–0001-5647–4297 Nikolai Norevik Myklebust, Department of Medicine, Haukeland University Hospital, Bergen, Norway Oddvar Oppegaard, Department of Clinical Science, University of Bergen, Department of Medicine, Haukeland University Hospital, Bergen, Norway Camilla Tøndel, Department of Clinical Science, University of Bergen, Department of Pediatrics, Haukeland University Hospital, Bergen, Norway Rebecca Jane Cox, Influenza center, Department of Clinical Science, University of Bergen, and Department of Microbiology, Haukeland University Hospital, Bergen, Norway Arild Iversen, Bergen Municipality, Department of Clinical Science, University of Bergen, Norway Marianne Emblem Lehmann, Department of Clinical Science, University of Bergen, Bergen, Norway Axel Sandvig, St Olav’s Hospital, Trondheim, and Department of Neuromedicine and Movement Science, Norwegian University of Science and Technology, Trondheim, Norway Tuva Børresdatter Dahl, Research Institute for Internal Medicine, Oslo University Hospital, Oslo, Norway Vibeke Devold Valderhaug, Department of Research and Innovation, Møre og Romsdal Hospital Trust, Ålesund, Norway, and Department of Neuromedicine and Movement Science, Faculty of Medicine, Norwegian University of Science and Technology, Trondheim, Norway Peter Szodoray, Department of Immunology, Oslo University Hospital—Rikshospitalet, Oslo, Norway. ORCID 0000–0003-4443–6159 Maja Wilhelmsen, University Hospital of Northern Norway, Tromsø, and Arctic University of Norway, Tromsø, Norway Bjørn Eivind Kirsebom, Department of Neurology, University Hospital of North Norway, Tromsø, and Department of Psychology, Faculty of Health Sciences, The Arctic University of Norway, Tromsø, Norway. ORCID 0000–0002-1413–9578 Marte Glambek, Department of Medicine, Haukeland University Hospital, Bergen, Norway Oddvar Kaarbøe, Department of Global Public Health and Primary Care, University of Bergen Pål Aukrust, Research Institute of Internal Medicine, Oslo University Hospital, Rikshospitalet, Oslo, Norway, and Institute of Clinical Medicine, University of Oslo, Oslo, Norway Rolv Terje Lie, Department of Global Public Health and Primary Care, University of Bergen. ORCID: 0000–0001-6287–4051 Nina Langeland, Department of Clinical Science, University of Bergen, National Centre for Tropical Infectious Diseases, Department of Medicine, Haukeland University Hospital, Bergen, Norway
Name and contact information for the trial sponsor {5b}	Professor Nina Langeland, Department of Clinical Science, University of Bergen. N5021 Bergen Norway. Email: nina.langeland@uib.no. Phone: + 47- 41,616,450
Role of sponsor {5c}	This is an investigator-initiated clinical trial without involvement of any pharmaceutical company. The study sponsor is Haukeland University Hospital, a publicly funded tertiary referral hospital in Western Norway. The investigator team is solely responsible for the study design; collection, management, analysis, and interpretation of data; writing of the report; and the decision to submit the report for publication. The investigators will publish the findings in reputable international scientific journals after completing the study

## Introduction

### Background and rationale {6a}

The COVID-19 pandemic has had a global impact with more than 750 million confirmed cases and an estimated 6.9 million deaths [1]. Even though the number of COVID-19 hospitalizations is decreasing, a considerable burden of long-term complications, referred to as long COVID, has been reported after acute SARS-CoV-2 infection [2–5]. The World Health Organization (WHO) classifies long COVID as the continuation of, or development of new symptoms 3 months after the initial SARS-CoV-2 infection, lasting for at least 2 months [6]. Long COVID appears to be a more common post-viral condition than illnesses after other viral infections [7]. Acute COVID-19 presenting with severe disease manifestations requiring hospitalization is particularly associated with long-term complications. Nevertheless, long COVID can occur in up to 30% of patients even after mild to moderate acute SARS-CoV-2 infection [2, 5, 8]. In line with this, the risk of developing long COVID after infection with an Omicron variant is similar to that after a Delta variant infection [9]. The duration of long COVID is unclear, but over 30% of sequelae persist at 2-year follow-up even in patients who had not been hospitalized during the initial infection [10]. The high prevalence of long-term persisting symptoms after COVID-19 may have a large public health impact [11]. The pathophysiology of long COVID is still unresolved, and there is still no cure for this condition [12]. Apart from avoiding infection, there are no definite treatment strategies for long COVID, although there is some evidence for the partial protective effect of leading a healthy lifestyle [13], vaccination [14, 15], and receiving antiviral treatment for acute COVID-19 [16]. Better preventive measures are needed. Early treatment with the novel antiviral combination nirmatrelvir/ritonavir (Paxlovid®) prevents hospitalization and death from COVID [17, 18]. In the current trial, we aim to assess whether treatment with nirmatrelvir/ritonavir for acute COVID-19 regardless of disease severity during the acute phase can prevent persistent symptoms or long COVID at 3-month follow-up and beyond.

### Objectives {7}

#### Hypothesis

Antiviral treatment of acute COVID-19 can prevent persisting symptoms compatible with long COVID at 3 months and beyond.

#### Specific objectives

The primary objective is to assess whether a 5-day course of nirmatrelvir/ritonavir (Paxlovid®), two tablets of 150 mg nirmatrelvir and one tablet of 100 mg ritonavir twice daily, for acute COVID-19 can reduce the prevalence of persistent symptoms at 3 to 24 months compared to placebo.

### Trial design {8}

PanoramicNOR is a two-arm 1:1 randomized clinical trial assessing whether adults (P—study population) treated with nirmatrelvir/ritonavir (I—intervention) for acute COVID-19 versus those treated with placebo (C—comparator) will have a reduced probability of suffering persistent symptoms at 3 months and beyond (O—outcome). This is a superiority study with parallel groups.

## Methods: participants, interventions and outcomes

### Study setting {9}

The main study site is the setting of municipal healthcare services in Bergen, Norway. Two additional inclusion sites are in Ålesund and Oslo, Norway. Follow-up of participants and assessment of adverse events are done by study personnel at the main study site in Bergen.

### Eligibility criteria {10}

#### Inclusion criteria

Individuals aged 18 to 65 years with symptoms of 0- to 5-day duration consistent with COVID-19, and a positive PCR or lateral flow test for SARS-CoV-2 from throat or nasopharyngeal swab taken between 2 days before symptom onset and the time of screening, are eligible to be included. Patients must be able and willing to provide informed consent. Women of childbearing potential will be offered a pregnancy test, and included if they are willing to take this test, and the test result is negative.

#### Exclusion criteria

Patients with symptom duration of more than 5 days will be excluded. Pregnant and lactating women will not be included. Persons will not be included if they, by judgement of the investigator, may need nirmatrelvir/ritonavir treatment as routine care because of immunosuppressive conditions or other comorbidities (such as malignancies). Patients with chronic renal impairment or chronic liver dysfunction will be excluded. Patients will be excluded if they use concomitant medication contraindicated for the treatment of Paxlovid (see list in supplementary information), unless the study physician can make a safe and agreeable plan for dose adjustment or temporary stopping of the medication in question. Patients will not be recruited if they lack the capacity to consent, if they indicate they are not able to complete all study visits, if they are currently taking nirmatrelvir/ritonavir, if they are allergic to nirmatrelvir/ritonavir, if they are currently admitted to hospital, if they have been randomized in this trial before, or if they are participating in another clinical trial of a therapeutic agent.

### Who will take informed consent? {26a}

Patients testing positive for COVID-19 attending the study recruitment office will be screened for eligibility by study staff, consisting of nurses and a medical doctor, who will also obtain informed consent.

Eligible participants will be asked to physically attend the study site for enrolment in the study. Written informed consent is provided after discussion between a member of the trial team and the participant, where the risks and benefits of taking part and follow-up procedures will be explained. Prior to consent, written and summary versions of the patient information sheet (PIS) and informed consent form (ICF) will be available to participants detailing the exact nature of the trial and the known side effects and risks involved in taking part. Participants will be informed that they are free to withdraw from the study at any time without giving any reasons. Adequate time will be given to the participant to consider the information and to ask any questions about the trial before deciding whether to participate. For participants who are too unwell or unable to respond to surveys themselves, a study partner who they identify will be able to assist the participant in completing screening, baseline, and follow-up online forms and/or calls and provide information to them on their behalf when necessary.

After informed consent is signed, participants will enter online baseline information, including demographics, comorbidities, concomitant medication, allergies, COVID-19 vaccine history, and previous COVID-19, residual symptoms of past COVID-19 infection, and their contact details and those of a study partner. Identifying a study partner is not a requirement to participate in the study.

### Additional consent provisions for collection and use of participant data and biological specimens {26b}

The trial team will use Norwegian health registries as source data for relevant medical information, including emergency hospitalization events (Norwegian Patient Registry—NPR), details concerning COVID-19-related hospital admissions (Norwegian Intensive Care and Pandemic Registry—NiPAR), mortality and cause of death (Norwegian Cause of Death Registry), contacts with primary care (Norwegian Registry for Primary Health Care—KPR), COVID-19 vaccine history (Norwegian Immunization Registry—SYSVAK), drug prescriptions (Norwegian Prescription Database—NorPD), and sick leave (The Sickness Absence Registry).

Data collected will include participant identifiable information and will be accessed at Haukeland University Hospital according to Primary Care Clinical Trials Unit (PC-CTU) Information Governance policies and Norwegian General Data Protection Regulations (GDPR). Data will only be held for the duration it is required; this will be reviewed annually. All documents will be stored safely and confidentially. On all study-specific documents, other than the signed consent, the participant will be referred to by the study participant number/code, not by name.

A subgroup of up to 500 patients will be asked to attend a face-to-face visit and/or to donate a microbiological or blood sample for the purpose of the study, at 3, 6, and 12 months after inclusion. A subset of 100 patients, with and without symptoms at 3 months, will be included in a study to characterize any effects on the brain by neurocognitive assessment, electroencephalography (EEG), and magnetic resonance imaging (MRI) investigations. The two subgroups of participants will have additional consent forms specific to the respective sub-studies.

## Interventions

### Explanation for the choice of comparators {6b}

At the initiation of the study, nirmatrelvir/ritonavir was the only antiviral drug on the market approved to treat COVID-19 in an outpatient setting in Norway. Currently, neither nirmatrelvir/ritonavir nor any other pharmacological agents have proven efficacy in preventing long COVID. Therefore, we have chosen to compare nirmatrelvir/ritonavir with placebo.

### Intervention description {11a}

Participants in the intervention arm will receive a standard 5-day treatment course of nirmatrelvir/ritonavir in addition to standard of care. Participants in the control arm will receive a 5-day course of placebo tablets, with the same appearance and quantity, in addition to standard of care. An independent professor at the University of Bergen, who was not part of the trial team, made a randomization list of 2000 numbers with 1:1, non-stratified randomization to interventional product or placebo. The company preparing placebo products coated both the interventional products and the placebos with identical coating and identical labelling of the package apart from the unique study number. Participants are consecutively allocated according to the pre-made randomization list to receive either the antiviral agent nirmatrelvir/ritonavir in addition to standard of care or placebo plus standard of care. They will receive the study medication in two containers marked “nirmatrelvir/placebo” and “ritonavir/placebo,” both with their study number on the box, and be given both oral and written instructions on how to take the study medication. The study medication will be taken at home at the same dosage as is recommended for nirmatrelvir/ritonavir, one dose (two tablets nirmatrelvir/placebo and one tablet ritonavir/placebo) morning and evening for 5 days. The study nurse or study doctor will call the participant between day 1 and day 3 to check if the participants are taking the study medication as prescribed and whether they have experienced any adverse reactions. After 5 days of taking the oral study medication, there are no further medical interventions in the study.

### Criteria for discontinuing or modifying allocated interventions {11b}

Each participant has the right to withdraw from the study at any time without giving reasons or justification. In addition, the investigator team may discontinue a participant from the study at any time if the investigator team considers it necessary for any reason, including ineligibility (either arising during the study or retrospectively) or withdrawal of consent. Data that has already been collected about the participant will be kept and included in the analyses of the trial. Withdrawn participants will not be replaced. Although withdrawals, in principle, may reduce the power of the study, we do not expect the attrition to be sufficient to make this a serious concern. If serious adverse events occur, study medication will be stopped, but the patient will be followed up and data collected. Only the standard dose of nirmatrelvir/ritonavir is used in this study, as per the manufacturer’s instructions.

### Strategies to improve adherence to interventions {11c}

A safety call will be made to all participants between days 1 and 3 to ensure compliance and that the medication is used as prescribed. Moreover, medication adherence will be captured daily in the electronic diaries or in phone calls with the patient. The research team will call participants/study partners who have not completed their diary for at least 2 days before day 7. No more than two contact attempts will be made at each of these follow-up points. Deviation(s) from the prescribed dosage regimen will be recorded. The participants not completing the intervention will be asked to return any unused intervention drugs to the research center for destruction.

### Relevant concomitant care permitted or prohibited during the trial {11d}

The study doctor will check relevant interactions between participants’ regular medicines and nirmatrelvir/ritonavir to ensure there are no serious known interactions before the participant is enrolled in the trial. Concomitant medications that are contraindicated for the treatment of nirmatrelvir/ritonavir include the following (supplemental information):

- Medicinal products that are highly dependent on the enzyme CYP3A for clearance and for which elevated concentrations are associated with serious and/or life-threatening reactions.
- Medicinal products that are potent CYP3A inducers where significantly reduced nirmatrelvir/ritonavir plasma concentrations may be associated with the potential for loss of virologic response and possible resistance.
- Nirmatrelvir/ritonavir cannot be started immediately after discontinuation of any of the medicinal products listed specifically in the protocol due to the delayed offset of the recently discontinued CYP3A inducer.
- Medicinal products that are contraindicated with nirmatrelvir/ritonavir according to the SmPC: https://www.ema.europa.eu/en/medicines/human/EPAR/paxlovid.

The study doctor on site will make a judgement and may adjust the patient’s regular medication for the intervention period (5 days of treatment + 7 days of residual effect of nirmatrelvir/ritonavir) to avoid drug–drug interactions, if this is deemed safe and the patient finds it acceptable.

### Provisions for posttrial care {30}

Patients are insured in accordance with the Product Liability Act in the Drug Insurance and the Patient Injuries Act.

### Outcomes {12}

A total of 13 symptoms will be recorded in the baseline questionnaire and all follow-up questionnaires (days 1–7, weeks 2, 3, and 4, and at 3, 6, 12, and 24 months, Table 1). The following symptoms are recorded as binary variables: fever, tingling sensation, headache, dizziness, altered taste/smell, sleep disturbances, chest pain, muscle/joint pains, and nausea. The following will be recorded as graded symptoms (better than usual, as usual, worse than usual, much worse than usual): fatigue, dyspnea, memory problems, concentration problems, and feeling depressed. Additionally, we will record absence from work/school (number of days), hospitalization (number of days), and any contact with a doctor/emergency clinic.

**Table 1:** Schedule of enrolment, interventions and assessments

TIMEPOINT	*STUDY PERIOD*															
	*ENROLMENT*	ALLOCATION	*POST-ALLOCATION*										*CLOSE-OUT*			
			*Days*							*Weeks*			*Months*			
	*	D0	*1*	*2*	*3*	*4*	*5*	*6*	*7*	*2*	*3*	*4*	*3*	*6*	*12*	*24*
ENROLMENT:																
Eligibility screen	X															
Informed consent	X															
* Pregnancy test†*	X															
Allocation		X														
INTERVENTIONS:																
* Paxlovid*		X	X	X	X	X										
* Placebo*		X	X	X	X	X										
ASSESSMENTS:																
* Baseline CRF*		X														
* Phone call to patient*			X	X	X											
* Daily eCRF*			X	X	X	X	X	X	X							
* Weekly eCRF*										X	X	X				
* Primary endpoint eCRF*													X			
* Follow-up eCRF*														X	X	X
* Compliance*			X	X	X	X	X									
* Adverse events‡*			X	X	X	X	X	X	X	X	X	X	X	X	X	X
SUBSTUDIES																
* Blood samples*		X											X	X	X	
* Sputum sample*		X														
* MRI scan*													X	X		
* EEG*													X	X		
* Neuropsychological evaluation*													X	X		

^*^Enrolment was done on the same day as allocation, or during previous days, as long as allocation could be done within 5 days of symptom start

^†^For women of childbearing potential

^‡^In addition to participants being questioned about adverse events in each eCRF throughout the study, they had option to spontaneously report adverse events at any time

#### Primary outcome

The primary outcome is a dichotomous variable for the presence of any of the three most important long COVID symptoms: (i) fatigue, (ii) dyspnea, and (iii) cognitive symptoms (defined as memory and/or concentration problems). The outcome is coded 1 for the presence of any one or more of these three symptoms and 0 for the absence of all the three symptoms. The primary outcome will first be evaluated at 3-month follow-up and then re-evaluated at 6-, 12-, and 24-monthsfollow-up.

Secondary outcomes include assessment of the intervention’s effect on the following:

- All individual symptoms separately and grouped by systems (systemic symptoms, chest-symptoms, cognitive, other neuropsychiatric symptoms).
- Graded responses for separate symptoms and symptom constellations, including an ordinal variable graded 0–3 for the presence of the three symptoms in the primary outcome.
- Analysis of potential risk factors for long COVID such as gender, age, other demographic characteristics, comorbidities, vaccination history, and use of any medications.
- Severity of acute disease using an eight-step scale, according to Beigel et al. [19]
- Hospitalization (binary).
- Mortality at 3 months (binary).
- Severe adverse events (binary).
- Absence from work, binary and graded by number of days.
- Societal cost/economic analysis, including estimated cost of absence from work/school, hospitalizations, deaths, quality-adjusted life years (QALYs) lost according to EQ-5D-5L, and more.

Subgroup analyses include the effect of intervention on the following:

- Immunological, inflammatory, and metabolic markers in relation to the presence and severity of long COVID and other described outcomes, as well as their relation to other factors such as demographic characteristics, comorbidities, vaccination history, and use of any medications.
- Brain pathology assessment by MRI, including functional MRI and tensor MRI, encephalography, and neuropsychological testing, in relation to the presence and severity of long COVID, as well as their relation to other described outcomes and factors such as demographic characteristics, comorbidities, vaccination history, and use of any medications.

### Participant timeline {13}

#### Sample size {14}

We expect to be able to recruit 2000 patients for randomization in this study. The presence of long COVID is our primary endpoint. Prior knowledge suggests that long COVID may be present among approximately 30–50% of the patients. With a total sample size of approximately 2000, we will be able to detect a 16–22% treatment effect with a power of 90% (5% significance level) depending on the prevalence of long COVID symptoms in the placebo group (Table 2).

**Table 2:** Sample size scenarios

90% power and 5% significance level, two-sided test of superiority				
Placebo*	Treatment*	Treatment effect	NNT	Total sample size
50%	42.5%	16%	14	1908
40%	33%	17.5%	15	2042
30%	23.5%	21.7%	16	2008

^*^Expected prevalence of primary outcome

#### Recruitment {15}

Potential participants can present directly to the trial team via the trial website or telephone. Dissemination of trial information and recruitment of potential trial participants will commence through several channels:

I. All health professionals (including primary care physicians and Test and Trace staff, pharmacy staff, etc.) will be able to provide information about participation and direct potential participants to the online trial information and the trial website.
II. Media campaigns will use television, radio, and social media platforms to generate awareness of the trial and to sign-post to the trial.
III. All facilities, including testing centers and municipality centers, will be able to inform potentially eligible participants about the clinical trial and refer them to the trial website and/or trial team.
IV. Clinicians can reach out to potentially eligible participants identified by receiving SARS-CoV-2 test results from Test and Trace and laboratories and by regular searches for patients with a positive SARS-CoV-2 test result in their clinical database. Contact can be made with potential participants verbally or by text, email, and telephone.

## Assignment of interventions: allocation

### Sequence generation {16a}

An independent scientist prepared a randomization list from 1 to 2000, assigning each number to treatment with nirmatrelvir/ritonavir or placebo using fixed equal allocation ratios. Upon inclusion in the study, participants are consecutively assigned to the next randomization number on the list and given the corresponding package of study product (either nirmatrelvir/ritonavir or placebo).

#### Concealment mechanism {16b}

The randomization list prepared by an independent scientist was given to the company producing the placebo capsules and used for packaging of interventional drugs and placebo. The active drugs and placebo products are coated so that they look identical, and packages appear identical. Packages are labeled with the ID number on the randomization list, and patients will be allocated this ID number.

#### Implementation {16c}

An independent scientist produced a randomization list, and drug packages were pre-randomized by a company accordingly. At the time of inclusion in the study, study personnel will receive the next study medication box and blindly allocate participants to receive the active ingredient or placebo according to the randomization list.

## Assignment of interventions: blinding

### Who will be blinded {17a}

PanoramicNOR is a double-blinded placebo-controlled trial. If unblinding or code breaking is required, as requested by the protocol or the Data and Safety Monitoring Board (DSMB), this will be made available to the DSMB. However, those managing the data will be blinded to participant allocation. The trial team and recruiting clinicians will be blinded to the results of interim analyses. During the course of the trial, only the unblinding statisticians and the independent members of the DSMB will have access to the unblinded interim results.

#### Procedure for unblinding if needed {17b}

If unblinding or code breaking is required, as requested by the protocol or the DSMB, this will be made available to the DSMB. However, those managing the data will remain blinded to participant allocation.

The trial team and recruiting clinicians will be blinded to emerging results of interim analyses. During the course of the trial, only the unblinding statisticians and the independent members of the DSMB will have access to the unblinded interim results.

The DSMB will recommend stopping a trial if as follows:

- There is a safety concern which warrants stopping the trial.
- The study will be stopped at the interim analysis if the proportion of deaths at 28 days follow-up is higher in one of the groups by a significance of *p* < 0.02 and effect size of odds ratio > 4. The strict significance level and high effect size was chosen to ensure the study would not be inappropriately terminated for random variation in hospitalizations and deaths. Given the inclusion of only patients with mild acute infection in a relatively healthy cohort < 65 years of age, we do not expect a high prevalence of severe outcomes.
- Severe adverse events will be reported consecutively to the DSMB. The DSMB will evaluate this independently of the interim analysis.

## Data collection and management

### Plans for assessment and collection of outcomes {18a}

As described under outcomes above, a total of 13 symptoms will be recorded, and the primary outcome is a dichotomous variable for the presence of any of the three most important long COVID symptoms: (i) fatigue, (ii) dyspnea, and (iii) cognitive symptoms (defined as memory and/or concentration problems). In addition, data on nine secondary outcomes will be collected, as described above. The data collection forms can be found at the end of the protocol.

### Plans to promote participant retention and complete follow-up {18b}

The participants will receive a text message with the questionnaire on each day they are supposed to answer a new form (daily: days 0–7, weekly: weeks 2–4, at 3, 6, 12, and 24 months). If they have not answered during the day, they will get a reminder in the evening to fill out the questionnaire. If the participant still has not answered the next day, the study team can make up to two phone calls to the participant to remind them to fill out the questionnaire form. After this, no further attempts to contact participants regarding the specific questionnaire form will be made, and missing data points will remain as such.

For the purpose of obtaining blood samples, the subgroup participants will be contacted in due time to schedule an appointment at the study center for follow-up blood tests. This will also be the case for the MRI group participants. In addition, all participants will get a phone call between days 1 and 3 to ensure they are taking nirmatrelvir/ritonavir or placebo correctly and answer any new questions that may have arisen since they were included.

### Data management {19}

The following summarizes the study’s data management plan.

#### Source data

Source documents are where data are first recorded. CRF entries will be considered source data where no other primary record is documented. The trial team may use Norwegian health registries mentioned under the “Additional consent provisions for collection and use of participant data and biological specimens {26b}” above as source data, including NPR, NiPAR, the Norwegian Cause of Death Registry, KPR, SYSVAK, NorPD, and the Sickness Absence Registry.

Data collected will include participant identifiable information and will be accessed at the Haukeland University Hospital/University of Bergen according to CTU information, governance policies, and Norwegian GDPR. Data will only be held for the duration it is required; this will be reviewed annually. All documents will be stored safely in confidential conditions. On all study-specific documents, other than the signed consent, the participant will be referred to by the study participant number/code, not by name.

#### Access to data

Direct access will be granted to authorized representatives from the sponsor, host institution, and the regulatory authorities to permit trial-related monitoring, audits, and inspections.

#### Data recording and record keeping

The investigators will maintain appropriate medical and research records for this trial, in compliance with the requirements of the Clinical Trials Regulation, no. 536/, ICH E6 GCP, and regulatory and institutional requirements for the protection of the confidentiality of volunteers. The chief investigator; principal investigator; co-investigators; clinical team, including study nurses; and other authorized members of the trial team will have access to records. The investigators will permit authorized representatives of the sponsor and regulatory agencies to examine (and when required by applicable law, to copy) clinical records for the purposes of quality assurance reviews, audits, and evaluation of the study's safety and progress.

The data will be entered into CRFs in an electronic format by the participant, trial partner, or trial team using the secure cloud-hosted SAFE server located at the University of Bergen, Norway. Data will be entered in a web browser and then transferred to the database by encrypted transfer. This includes safety data, laboratory data, and outcome data. Safety data will be collected through electronic diaries. Risks are mitigated using the ISO97001 framework.

The online secure data entry system REDCap (Research Electronic Data Capture, Vanderbilt University) will be used to collect study data. All identifiable participant data will be stored in a separate database in REDCap. The participant portal will also manage electronic patient-reported outcome data. Participant and trial partner data will be kept and stored securely for as long as it is required by the study and reviewed on an annual basis.

### Confidentiality {27}

Data with identifying variables will be stored on firewall-protected secure servers, separately from non-identifiable information such as clinical and microbiological data. Consent forms from participants will be safely stored separately from the collected data in a locked cabinet.

### Plans for collection, laboratory evaluation and storage of biological specimens for genetic or molecular analysis in this trial/future use {33}

Blood will be sampled at inclusion from the willing participants at the study sites. The blood will be sampled in 10-mL collecting tubes (2 × serum, 2 × EDTA, 4 × sodium heparin, and 1 × tempus blood RNA tube) using standard venipuncture technique. After collection, the blood samples will be handled in accordance with the blood sample SOP 1; further details can be found there. The serum and EDTA tubes will be frozen at minus 80 °C and the tempus blood RNA tube at minus 20 °C. The sodium heparin tubes will be used for whole blood preservation in cytodelics whole blood stabilizer solution and for peripheral blood mononuclear cells isolation; these procedures are described in blood sample SOP 2. We will also collect separate samples for CRP, D-dimer, and fibrinogen at inclusion and send these to the clinical laboratory at Haukeland University Hospital for storage and analysis. Blood to be sampled at 3-, 6-, and 12-month follow-up is detailed in blood sample SOP 3. Throat PCR swabs of all participants will be collected upon inclusion and frozen at minus 80 °C for later use.

## Statistical methods

### Statistical methods for primary and secondary outcomes {20a}

The primary outcome, i.e., the effect of intervention on a binary variable for the presence of any of three key long COVID symptoms, will be analyzed with an appropriate logistic regression model. Depending on whether we obtain a good balance of prognostic factors between the randomized groups, we may consider adjustment for prognostic factors in the analyses. We will report an odds ratio with 95% confidence intervals and a *p*-value from this analysis.

The primary analysis will be an “intention-to-treat” analysis performed on all patients who were randomized. In addition, a per-protocol analysis will be performed for patients who reported having adhered to the ingestion of the study product (antiviral or placebo) and on other subgroups of the study population. If adherence is incomplete, we will attempt to estimate the biological effect of the medication by using instrumental variable analysis [20].

In the analysis of follow-up data up to 24 months, we will use appropriate mixed models to account for the correlation of symptoms over time. We will estimate the main effect of treatment and test whether the effect is uniform over time.

Secondary outcomes will be analyzed with appropriate regression models depending on the type of outcome variable. For binary outcomes, we will use logistic regression. For the number of symptom counts, we will consider negative binomial regression, and for score outcomes, we will attempt ordinal logistic regression and quantile regression. Using quantile regression models, we will estimate the effect of the intervention also in the tails of the score distribution.

Further analysis plans include cost-efficacy analyses and explorative analyses. We will estimate the resource inputs associated with providing the antiviral treatment in routine clinical practice. Societal costs will be estimated using data on primary care encounters, hospital inpatient/day case admissions, outpatient visits, and accident and emergency attendances. Loss of quality-adjusted life year (QALY) will be estimated. Unit costs will be valued using national reference tariffs. Compound total health care cost per trial participant over the trial time horizon will be estimated. Secondary expressions of cost-effectiveness will include incremental cost per hospitalization and/or death prevented over 28 and 60 days. Relevant data may be obtained from abovementioned registries, including NPR, NIPaR, the Norwegian Cause of Death Registry, KPR, NorPD, SYSVAK, and the Sickness Absence Registry. Cost-effectiveness will be expressed in terms of incremental cost per QALY gained. Bivariate regression of costs and measures of health consequence, with multiple imputation of missing data, will be conducted to generate within-trial estimates of incremental cost-effectiveness. Sensitivity analyses will assess the impact of areas of uncertainty surrounding components of the economic evaluation. The probability of cost-effectiveness at alternative thresholds will be measured. Cost-effectiveness threshold values will be informed by guidance from government departments on the value placed by decision-makers on an additional QALY and on a statistical life.

### Interim analyses {21b}

Interim analyses will be performed when 1000 patients have reached 28 days follow-up, assessing any deaths and hospitalizations occurring in the study population, or at the request of the DSMB. The study will be stopped if the proportion of deaths at 28-day follow-up is higher in one of the groups by a significance of *p* < 0.02 and an effect size of odds ratio > 4. Severe adverse events will be reported consecutively to the DSMB. The DSMB will evaluate this independently of the interim analysis.

### Methods for additional analyses (e.g., subgroup analyses) {20b}

#### Study on brain sequelae in long COVID

##### Hypothesis

Neuroimaging can identify brain structural or functional damage in long COVID patients that correlate with neuropsychological deficits. This project aims to include 100 consecutive patients willing to participate in this sub-study. The objective of the sub-study is to document the hypothesized relationship between long COVID, neurocognitive dysfunction, and alterations in the brain neural connectome of patients with acute SARS-CoV-2 infection at 3- and 6-month time points. The exploratory secondary endpoints will be (i) loss of brain volume assessed with volumetric MRI, (ii) reduced resting state activity evaluated with fMRI, (iii) quantified reduction in the neural connectome using diffusion tensor MRI, (iv) persistent slowing of brain activity registered with EEG in the alfa and theta frequencies at rest and during cognitive tasks, and (v) quantification of cognitive domain functions with neuropsychological assessment. The study will aim to document deficits in memory, spatial navigation, cortical motor control, concentration and focus control, mental endurance, and affective processing.

#### Immune, inflammatory and metabolic profiling of patients with and without long COVID

##### Hypothesis

Long COVID appears to be a state of immune dysfunction that involves persistent inflammation, and the identification of biological, immune, and inflammatory parameters of this dysfunction will reveal potential additional targets for intervention, risk prediction, and prevention.

##### Methods

Biological samples such as blood and respiratory samples will be collected during the acute phase and convalescent phase at appropriate time periods with possible follow-up to 12 months. From the same group of 100 patients in subproject 1, supplemented with up to 400 additional patients to a total of a maximum of 500 participants, follow-up blood samples will be collected to allow detailed immune profiling, including 2 × 10 mL for serological investigations, EDTA 2 × 10 mL, proteomic sodium heparin 4 × 10 mL for peripheral blood mononuclear cells, and Tempus Blood RNA Tube 1 × for RNA analyses. We will correlate SARS-CoV-2-specific B- and T-cell functions and changes in inflammatory, immune, and metabolic markers with the presence and absence as well as the severity of long COVID. We will conduct state-of-the-art immunological assays using established protocols to investigate SARS CoV-2-specific binding and neutralizing antibodies to SARS-CoV-2 variants using ELISA and neutralization assays in SARS-CoV-2 antigen-specific B lymphocytes. Markers of immune activation/cellular exhaustion, cellular senescence/accelerated aging, extracellular matrix remodeling, vascular inflammation, and markers of impaired blood–brain-barrier function will be measured by high-throughput ELISA in all participants during follow-up. Proteomic analyses in serum plasma and RNA analyses and targeted/untargeted proteomic (mass spectrometry) on immune cells will be used as discovery platforms, and other analyses of immune cells/whole blood will include analyses of DNA repair mechanisms and mitochondrial function as well as epigenetic and epitranscriptomic modifications. SARS-CoV-2 antigen-specific T-cell responses, including memory and exhaustion markers, and SARS-CoV-2 spike-specific CD4 + T cells will be assessed by ELISpot and multiparametric flow cytometry, deep phenotyping of leukocyte subsets by mass cytometry, including T and B cells, monocytes, dendritic cells, and NK cell subsets.

### Methods in analysis to handle protocol nonadherence and any statistical methods to handle missing data {20c}

Any deviations from the protocol will be documented in a protocol deviation form and filed in the study master file. The Norwegian Clinical Research Infrastructure Network (NorCRIN) SOP is used to identify noncompliances, escalation to the central team, and assessment of whether a noncompliance/deviation may be a potential serious breach. Missing data for both binary and continuous variables will, in the main analyses, be handled as nonresponses. Depending on the degree of missingness, we may perform supplementary analyses with multiple imputation of missing values to assess the possibility that the missing data may introduce bias.

### Plans to give access to the full protocol, participant level-data and statistical code {31c}

The R code for the statistical analyses will be published alongside the articles from the study. Anonymous data from the study may be made available upon reasonable request.

## Oversight and monitoring

### Composition of the coordinating center and trial steering committee {5d}

The Trial Steering Committee (TSC) will ensure the rights, safety, and wellbeing of the trial participants. They will make recommendations about how the study is operating, any ethical or safety issues, and any data being produced from other relevant studies that might impact the trial. Composition and roles and responsibilities of the TSC are detailed in the TSC charter. The TSC advises the Trial Management Group (TMG) about the conduct of the study and stopping randomization to study arms (based on recommendations received from the DSMB and/or relevant information external to the trial). If national recommendations in standard of care change during the study period, the TSC will make recommendations to the TMG regarding potential new risks or benefits. The Statistical Analysis Committee (SAC) will perform interim analyses and report these to the DSMB. The TMG will remain blind to these interim analyses until a recommendation is received from the TSC about stopping randomization or safety concerns. TMG will be responsible for the day-to-day running of the trial, including monitoring all aspects of the trial and ensuring that the protocol is being adhered to. It will include co-investigators and will meet weekly in the first instance. Composition, roles, and responsibilities of the TMG are detailed in the TMG charter. A core project team (PT) from within the TMG will meet weekly or as required for operational decision-making.

### Composition of the data monitoring committee and its role and reporting structure {21a}

The DSMB will review the data received from the SAC at each interim analysis, as described in the statistical analysis section, in order to ensure that the process is working correctly and to review and monitor the accruing data to ensure the rights, safety, and wellbeing of the trial participants. The composition, roles, and responsibilities of the DSMB are detailed in the DSMB charter. The DSMB reviews data from interim analyses and makes recommendations to the TSC if safety concerns have emerged.

### Adverse event reporting and harms {22}

The following summarizes the handling of potential adverse events (AE). The detailed plan can be found in the study protocol.

#### Collecting AE

Symptoms, adverse events, and serious adverse events (SAE) will be collected from participants’ daily diaries, as well as calls to participants/study partners. All participants will receive a call on days 1–3 to make sure that they understand the possible risks associated with nirmatrelvir/ritonavir and how to report potential side effects and seek medical care if required. Participants will be provided with a 24-h contact telephone line to report any AEs that they experience and are concerned about, directly to a clinician. We will collect symptoms and side effects from symptom diaries and participant telephone calls.

#### Assessing AE

AEs will be monitored from the start of treatment for 12 months and assessed by a clinician (independent from the sponsor) for causality and severity. The study personnel on site will go through each new diary entry daily on weekdays to assess potential AEs and grade these from mild to severe. Also, the DMSB will review weekly reports of unblinded symptom data to identify potential side effects of nirmatrelvir/ritonavir. Any safety signals will be communicated to the TSC and TMG. The TMG will monitor SAEs and AEs and calls to the 24-h safety phone line for potential safety signals.

#### Reporting AEs

An annual report will be developed and submitted within 60 days after the anniversary date that the trial received clinical trial authorization. Due to the nature of this trial and the importance of sharing the science of COVID-19 and this novel drug, we expect to produce reports to the Norwegian government and regulatory agency more frequently upon request.

#### Reporting SAEs and SUSARs

An investigator will review the SAE once reported, collect as much information as needed, and report to the sponsor delegate within the timeframe according to the NorCRIN’s standard operating procedures (SOPs). The investigator will make the assessment of causality. AEs/SAEs judged possibly, probably, or definitely related will be considered as related to the antiviral agent. SAEs must be reported to the sponsor by the person who has discovered the SAE or a nominated delegate within 24 h of becoming aware of the event. The sponsor or delegate will ensure it is reviewed by the medical monitor or other delegated personnel for relatedness and expectedness as soon as possible to report any potential SUSAR to the relevant competent authority. For SAEs that require reporting, the expectedness of SAEs will be assessed and determined by the medical monitor who is unblinded, according to the relevant Reference Safety Information (RSI) section of the Summary of Product Characteristics (SmPC). The RSI will be the current Norwegian Medical Products Agency (NoMA)/European Medicines Agency (EMA)-approved SmPC version at the time of the event occurrence. All SUSARs will be reported by the sponsor delegate to the relevant competent authority and other parties as applicable.

### Frequency and plans for auditing trial conduct {23}

The first monitoring at each site will be scheduled after the inclusion of the first 10 study subjects. The sites will be monitored on-site every other month in the first half of the year and then yearly according to the number of trial subjects included in the study. Additional monitoring will be done if requested by the DSMB. If needed or in case of concerns, monitoring of patients at St. Olav Hospital may occur. The monitoring plan will be updated if applicable. At the monitor visit, source data verification (SDV) will be performed for the following data.

#### Informed consent

- Check the first 100 included patients informed consent form and then 100 informed consent forms at a random selection during the inclusion period. In total, there were 10% of all signed informed consent forms.
- Check the first 50 informed consent forms for patients participating in the sub-study: Study on brain damage in long COVID.

#### AE/SAE/SUSAR

- Check for AE/SAE/SUSARs at patient records and eCRF are reported according to protocol.

#### DSMB meetings

- The monitor will check at each monitoring that DSMB meetings have been held according to DSMB charter by checking a statement letter written by the committee to ensure that the meeting has been held according to plan.

### Plans for communicating important protocol amendments to relevant parties (e.g., trial participants and ethical committees) {25}

Any substantial amendments to the protocol will be submitted to the relevant medical research ethical committee.

### Dissemination plans {31a}

Findings on the primary outcome will be published as soon as possible after the completion of the study in a high-rank peer-reviewed journal with open access. Publications on secondary outcomes will be published in due time.

## Discussion

As 750 million people have suffered COVID-19, the burden of long COVID is substantial. Apart from avoiding infection, we have few remedies to avoid long COVID. This study is unique in investigating whether treatment with nirmatrelvir/ritonavir during acute COVID-19 can reduce the risk of experiencing long COVID. A limitation to the blinding of the intervention is the characteristic taste of nirmatrelvir-ritonavir, which may lead participants to suspect that they have received active treatment and not placebo [21]. However, since participants are recruited during acute symptomatic COVID-19, and most participants will know that taste and smell disturbances are common in COVID-19, they may also ascribe the taste to the illness itself. If the study results were to confirm an effect of nirmatrelvir/ritonavir to prevent long COVID, this would be a major breakthrough in preventing the burden of this emerging health problem. The results of this study may also lead to improved understanding of the mechanisms underlying long COVID. The sub-studies on neurological aspects and immune profiling can provide further insights into the mechanisms and resultant pathology of long COVID. Cost–benefit analysis will contribute to increased understanding of the societal burden of long COVID and the potential benefits of treatment for this ailment.

### Trial status

The study protocol is version 2.3, dated 23rd March 2023. The first patient was recruited on the 12th of May 2023. The study is ongoing, and recruitment is estimated to be complete in December 2025.

## Supplementary Information

Additional file 1. Panoramic study: List of medicines that are contraindicated with concomitant use of nirmatrelvir/ritonavir.

## Data Availability

R-code used for data analysis will be made available with the published manuscript. The database will not be made openly available due to patient confidentiality, but anonymized data may be made available to researchers upon reasonable request.

## Abbreviations

SARS-CoV-2: Severe acute respiratory syndrome coronavirus 2
COVID-19: Coronavirus disease 2019
WHO: World Health Organization
DSMB: Data and safety monitoring board
IMP: Investigational medicinal product
CRF: Case report form
SDV: Source data verification
AE: Adverse event
SAE: Serious adverse event
SUSAR: Suspected unexpected serious adverse reaction
SOP: Standard operational procedure
RSI: Reference Safety Information
NorCRIN: Norwegian Clinical Research Infrastructure Network
NoMA: Norwegian medicinal product agency
EMA: European medicine agency
SmPC: Summary of product characteristics
PIS: Patient information sheet
ICF: Informed consent form
GDPR: General data protection regulations
PC-CTU: Primary care clinical trials unit
QALY: Quality-adjusted life years
