# Supplementary material for: Randomized trial of nirmatrelvir/ritonavir versus placebo for adults with acute COVID-19 to prevent long COVID: PanoramicNOR Trial
Source: Trials. 2025 Nov 6;26:477. doi: 10.1186/s13063-025-09226-6 (PMC12590622; doi:10.1186/s13063-025-09226-6)
Supplement: Supplementary file 1 — Additional file 1. Panoramic study: List of medicines that are contraindicated with concomitant use of nirmatrelvir/ritonavir. [file 13063_2025_9226_MOESM1_ESM.pdf]

Panoramic study

List of medicines that are contraindicated with concomitant use of nirmatrelvir/ritonavir

Alpha1-adrenoreceptor antagonist: alfuzosin
Analgesics: pethidine, piroxicam, propoxyphene
Antianginal: ranolazine
Anticancer drugs: neratinib, venetoclax
Antiarrhythmic: amiodarone, bepridil, dronedarone, encainide, flecainide, propafenone, quinidine
Antibiotics: fusidic acid, rifampicin
Anticonvulsants: carbamazepine, phenobarbital, phenytoin
Anti-gout: colchicine • Antihistamines: astemizole, terfenadine
Antipsychotics/neuroleptics: lurasidone, pimozide, clozapine, quetiapine
Ergot derivatives: dihydroergotamine, ergonovine, ergotamine, methylergonovine
GI motility agents: cisapride
Herbal products: St. John’s wort (Hypericum perforatum)
Lipid-modifying agents: o HMG Co-A reductase inhibitors: lovastatin, simvastatin o Microsomal triglyceride transfer protein (MTTP) inhibitor: lomitapide
PDE5 inhibitor: avanafil, sildenafil, vardenafil
Sedative/hypnotics: clorazepate, diazepam, estazolam, flurazepam, oral midazolam and triazolam
